# Supplementary material for: Unveiling the Bioactive Potential of the Invasive Jellyfish Phyllorhiza punctata Through Integrative Transcriptomic and Proteomic Analyses
Source: Biomolecules. 2025 Aug 4;15(8):1121. doi: 10.3390/biom15081121 (PMC12383608; doi:10.3390/biom15081121)
Supplement: Supplementary file 1 [file biomolecules-15-01121-s001.zip › Suplementary Material/FileS3/PEPFOLD4/XP_065057313_5407/clusters.html]

 PEP-FOLD clusters


Download archive of all models  here   

|  |  |  |
| --- | --- | --- |
| # | file | sOPEP |
| # cluster 1 - Size: 1 |  |  |
|  | XP\_065057313\_5407-model1 | -14.9112 |
| # cluster 2 - Size: 1 |  |  |
|  | XP\_065057313\_5407-model2 | -11.3163 |
| # cluster 3 - Size: 1 |  |  |
|  | XP\_065057313\_5407-model3 | -9.94655 |
| # cluster 4 - Size: 2 |  |  |
|  | XP\_065057313\_5407-model4 | -9.93684 |
|  | XP\_065057313\_5407-model4.1 | -9.60604 |
| # cluster 5 - Size: 6 |  |  |
|  | XP\_065057313\_5407-model5 | -9.83388 |
|  | XP\_065057313\_5407-model5.1 | -9.43063 |
|  | XP\_065057313\_5407-model5.2 | -9.42424 |
|  | XP\_065057313\_5407-model5.3 | -9.3513 |
|  | XP\_065057313\_5407-model5.4 | -9.22069 |
|  | XP\_065057313\_5407-model5.5 | -9.20185 |
| # cluster 6 - Size: 7 |  |  |
|  | XP\_065057313\_5407-model6 | -9.81054 |
|  | XP\_065057313\_5407-model6.1 | -9.79276 |
|  | XP\_065057313\_5407-model6.2 | -9.73716 |
|  | XP\_065057313\_5407-model6.3 | -9.6359 |
|  | XP\_065057313\_5407-model6.4 | -9.6336 |
|  | XP\_065057313\_5407-model6.5 | -9.43089 |
|  | XP\_065057313\_5407-model6.6 | -9.13027 |
| # cluster 7 - Size: 11 |  |  |
|  | XP\_065057313\_5407-model7 | -9.737 |
|  | XP\_065057313\_5407-model7.1 | -9.68044 |
|  | XP\_065057313\_5407-model7.2 | -9.42435 |
|  | XP\_065057313\_5407-model7.3 | -9.40925 |
|  | XP\_065057313\_5407-model7.4 | -9.40772 |
|  | XP\_065057313\_5407-model7.5 | -9.34023 |
|  | XP\_065057313\_5407-model7.6 | -9.31577 |
|  | XP\_065057313\_5407-model7.7 | -9.25338 |
|  | XP\_065057313\_5407-model7.8 | -9.18138 |
|  | XP\_065057313\_5407-model7.9 | -9.0987 |
|  | XP\_065057313\_5407-model7.10 | -8.96586 |
| # cluster 8 - Size: 25 |  |  |
|  | XP\_065057313\_5407-model8 | -9.7034 |
|  | XP\_065057313\_5407-model8.1 | -9.68535 |
|  | XP\_065057313\_5407-model8.2 | -9.66131 |
|  | XP\_065057313\_5407-model8.3 | -9.65039 |
|  | XP\_065057313\_5407-model8.4 | -9.64423 |
|  | XP\_065057313\_5407-model8.5 | -9.63128 |
|  | XP\_065057313\_5407-model8.6 | -9.62846 |
|  | XP\_065057313\_5407-model8.7 | -9.62157 |
|  | XP\_065057313\_5407-model8.8 | -9.55207 |
|  | XP\_065057313\_5407-model8.9 | -9.51443 |
|  | XP\_065057313\_5407-model8.10 | -9.48338 |
|  | XP\_065057313\_5407-model8.11 | -9.43316 |
|  | XP\_065057313\_5407-model8.12 | -9.40391 |
|  | XP\_065057313\_5407-model8.13 | -9.39023 |
|  | XP\_065057313\_5407-model8.14 | -9.38816 |
|  | XP\_065057313\_5407-model8.15 | -9.38595 |
|  | XP\_065057313\_5407-model8.16 | -9.30527 |
|  | XP\_065057313\_5407-model8.17 | -9.28312 |
|  | XP\_065057313\_5407-model8.18 | -9.20538 |
|  | XP\_065057313\_5407-model8.19 | -9.1968 |
|  | XP\_065057313\_5407-model8.20 | -9.18799 |
|  | XP\_065057313\_5407-model8.21 | -9.18763 |
|  | XP\_065057313\_5407-model8.22 | -9.185 |
|  | XP\_065057313\_5407-model8.23 | -9.13251 |
|  | XP\_065057313\_5407-model8.24 | -8.93294 |
| # cluster 9 - Size: 4 |  |  |
|  | XP\_065057313\_5407-model9 | -9.65195 |
|  | XP\_065057313\_5407-model9.1 | -9.52189 |
|  | XP\_065057313\_5407-model9.2 | -9.46903 |
|  | XP\_065057313\_5407-model9.3 | -9.37663 |
| # cluster 10 - Size: 1 |  |  |
|  | XP\_065057313\_5407-model10 | -9.58073 |
| # cluster 11 - Size: 21 |  |  |
|  | XP\_065057313\_5407-model11 | -9.57439 |
|  | XP\_065057313\_5407-model11.1 | -9.56802 |
|  | XP\_065057313\_5407-model11.2 | -9.54534 |
|  | XP\_065057313\_5407-model11.3 | -9.45219 |
|  | XP\_065057313\_5407-model11.4 | -9.44661 |
|  | XP\_065057313\_5407-model11.5 | -9.40988 |
|  | XP\_065057313\_5407-model11.6 | -9.38585 |
|  | XP\_065057313\_5407-model11.7 | -9.37114 |
|  | XP\_065057313\_5407-model11.8 | -9.30352 |
|  | XP\_065057313\_5407-model11.9 | -9.30029 |
|  | XP\_065057313\_5407-model11.10 | -9.28938 |
|  | XP\_065057313\_5407-model11.11 | -9.26639 |
|  | XP\_065057313\_5407-model11.12 | -9.26515 |
|  | XP\_065057313\_5407-model11.13 | -9.26234 |
|  | XP\_065057313\_5407-model11.14 | -9.25602 |
|  | XP\_065057313\_5407-model11.15 | -9.25066 |
|  | XP\_065057313\_5407-model11.16 | -9.22246 |
|  | XP\_065057313\_5407-model11.17 | -9.08279 |
|  | XP\_065057313\_5407-model11.18 | -9.06305 |
|  | XP\_065057313\_5407-model11.19 | -9.00244 |
|  | XP\_065057313\_5407-model11.20 | -8.84348 |
| # cluster 12 - Size: 5 |  |  |
|  | XP\_065057313\_5407-model12 | -9.56179 |
|  | XP\_065057313\_5407-model12.1 | -9.44055 |
|  | XP\_065057313\_5407-model12.2 | -9.37684 |
|  | XP\_065057313\_5407-model12.3 | -9.34536 |
|  | XP\_065057313\_5407-model12.4 | -9.23263 |
| # cluster 13 - Size: 2 |  |  |
|  | XP\_065057313\_5407-model13 | -9.50642 |
|  | XP\_065057313\_5407-model13.1 | -9.10447 |
| # cluster 14 - Size: 4 |  |  |
|  | XP\_065057313\_5407-model14 | -9.47325 |
|  | XP\_065057313\_5407-model14.1 | -9.17475 |
|  | XP\_065057313\_5407-model14.2 | -9.04283 |
|  | XP\_065057313\_5407-model14.3 | -8.91081 |
| # cluster 15 - Size: 3 |  |  |
|  | XP\_065057313\_5407-model15 | -9.38238 |
|  | XP\_065057313\_5407-model15.1 | -9.32184 |
|  | XP\_065057313\_5407-model15.2 | -9.17143 |
| # cluster 16 - Size: 1 |  |  |
|  | XP\_065057313\_5407-model16 | -9.37417 |
| # cluster 17 - Size: 3 |  |  |
|  | XP\_065057313\_5407-model17 | -9.26684 |
|  | XP\_065057313\_5407-model17.1 | -9.23158 |
|  | XP\_065057313\_5407-model17.2 | -9.227 |
| # cluster 18 - Size: 2 |  |  |
|  | XP\_065057313\_5407-model18 | -9.01858 |
|  | XP\_065057313\_5407-model18.1 | -8.88913 |
|  |  |  |
